# Antinociceptive, Anti-Inflammatory and Acute Toxicity Effects of Juglans Regia L. Leaves in Mice

**Published:** 2011-01-01

**Authors:** H Hosseinzadeh, H Zarei, E Taghiabadi

**Affiliations:** 1 Pharmaceutical Research Center, Department of Pharmacodynamy and Toxicology, School of Pharmacy, Mashhad, Iran; 2 Department of Pharmacodynamy and Toxicology, School of Pharmacy, Mashhad University of Medical Sciences, Mashhad, Iran

**Keywords:** Juglans regia, Hot-plate test, Writhing test, Xylene induced ear edema test, Cotton pellet test, Mice

## Abstract

**Background:**

Juglans regia leaves have been used in folk medicine to alleviate inflammatory diseases. This study investigates the antinociceptive, anti-Inflammatory and acute toxicity effects of Juglans regia L. leaves in mice.

**Methods:**

351 Male and female albino mice were divided into negative (saline), positive (morphine or diclofenac) controls as well as test groups (n=6-8). The acute (intraperitoneally) toxicity was evaluated for 2 days. Antinociceptive activities were done using hot-plate and writhing tests. Anti-inflammatory effects were studied using xylene induced ear edema and cotton pellet tests.

**Results:**

The LD50 values of J. regia aqueous and ethanolic extrats were 5.5 and 3.3 g/kg, respectively. The aqueous (2.87 and 1.64 g/kg) and ethanolic (2.044 and 1.17 g/kg) extracts showed antinociceptive activity in hot-plate test. The pretreatment of naloxone (2 mg/kg, s.c.) did not inhibit the extracts activities. The extracts exhibited antinociceptive activity in writhing test, which were not blocked by naloxone. In xylene test, both extracts showed anti-inflammatory activity in some doses. The extracts showed anti-inflammatory activity against the chronic inflammation.

**Conclusion:**

J. regia leaves demonstrated antinociceptive effect through non-opioid receptors and anti-inflammatory effect against acute and chronic inflammation. The extracts of J. regia could be considered as a promising analgesic and anti-inflammatory agents against diseases such as rheumatoid arthritis.

## Introduction

The Juglandaceae family has eight genera and the best-known species is the walnut, Juglans regia, which produces timber and edible nuts.[1] J. regia L. (Juglandaceae), is cultivated around the world such as in the West Indies, Japan, and South of Asia, in South Eastern Europe and in the eastern and southern region of the United States.[2]

Green walnuts, shells, kernels, bark and leaves have been used in the pharmaceutical and cosmetic products.[3] The leaves and pericarp of J. regia have been used as extracts in traditional medicine and pharmacologically demonstrated to be anti-helmintic, astringent, antifungal, hypoglycaemic, antidiarrhoeal and more recently, sedative.[1] Phenolic compounds are secondary metabolites, which are reported to occur in abundance in fresh J. regia leaves. Flavonoids and naphthoquinones are the main phenolic compounds in walnut leaves.[4][5][6] Pain and inflammatory are the most common disorders alleviated with folk and traditional medicine. Therefore, it is important to investigate the potential of herbal medicine for the discovery of new bioactive drugs.[7] The antinociceptive and anti-inflammatory effects of some plants such as, Zhumeria majdae, Cistus laurifolius, Elaeagnus angustifolia, Mentha piperita, Mentha pulegium, Crocus sativus, Salvia leriifolia Benth., Zataria multiflora and Verbascum salviifolium Boiss which have flavonoids constituents have been reported previously.[8]-[18] J. regia is used in folk medicine.[19] The poultice that is prepared from the stem bark of J. regia is used to treat inflammation in north east Italy,[20] and J. regia leaves are used for rheumatic pain in human adult in Turkey.[21]

There are a few studies on the anti-inflammatory and analgesic activity of this plant. The antinociceptive and anti-inflammatory effects of J. regia leaves were studied only in one dose in Turkey. In this study, the mechanism of antinociceptive activity (central or peripheral) and relevance with opioid receptors were not evaluated.[7] In another report, only antinociceptive activity of the ethanolic extract of J. regia leaves was demonstrated but anti-inflammatory effects and the mechanism of antinociceptive activity were not determined.[22]

The present study was undertaken to evaluate the anti-inflammatory, antinociceptive activity and acute toxicity of J. regia aqueous and ethanolic leaves extracts in different doses. Also the preliminary mechanism of antinociceptive effect (central or peripheral) and its correlation with opioid receptors would be evaluated.

## Materials and Methods

Fresh leaves of J. regia were collected from Avesina Reaserch Center of Mashhad (Khorassan Province), Northeastern Iran and were identified by Ferdowsi University. The voucher samples were preserved for reference in the herbarium of the Department of Pharmacognosy, School of Pharmacy, Mashhad (Voucher no. 146-1918-1). Naloxone hydrochloride and morphine sulfate were purchased from Tolid Daru Co., Tehran, Iran, diclofenac sodium from Darou Pakhsh Holding Co., Tehran, Iran. Xylene, acetic acid and chloroform were bought from Merk Co., Germany. Ampicilin vial was provided from Jaber Ebne Hayyan Pharmacy Co., Tehran, Iran and Ketamine was obtained from Trittau Co., Germany. All other chemicals and solvents used throughout this study were of analytical grade.

Three hundred and fifty one male and female albino mice (25±2 g each) were obtained from a randomly breed colony maintained on special diet in the animal house of Mashhad University of Medical Sciences. Animals were housed in a colony room under a 12/12 h light/dark cycle at 21±2 °C and had free access to water and food. The handling and use of animals were in accordance to the institutional guidelines and all experiments were carried out in accordance with current guidelines for the care of laboratory animals and the ethical guidelines on the use of animals (No:1024).

Animals of either sex were divided into several groups (n= 6-8). The first group received saline (10 ml/kg, i.p.) as negative control group. The groups that received diclofenac (15 mg/Kg, i.p.) and morphine (10 mg/Kg, i.p.) were considered as positive control for antinociceptive and anti-inflammatory tests, respectively. Based on maximum tolerated dose (MTD) of the aqueous (4.1 g/kg) and ethanolic (2.92 g/kg) extracts and 0.7, 0.4 and 0.1% of MTD, other groups received the aqueous extract at doses 0.41, 1.64 and 2.87 g/Kg, (i.p.) and the ethanolic extracts at doses 0.292, 1.17 and 2.044 g/Kg, (i.p.) as experimental groups. In cotton pellet test, higher dose of extracts were not injected for 7 days. The finale group was pretreated with naloxone (2 mg/Kg) by subcutaneous injection, 20 min prior to i.p. injection of the extracts and morphine.

Fresh leaves of J. regia were cleaned, dried in shadow and powdered by mechanical grinder. Then, the leaves powder (100 g) were defatted with petroleum ether (40-60°C) using the soxhlet apparatus. The powder was subsequently macerated in 500 ml ethanol (85%, v/v) for 3 days and the mixture was subsequently filtered and concentrated in vacuo at 40°C. The residue was suspended in saline. For the aqueous leaves extract, 1000 ml hot water was added to 100 g leaves powder, boiled for 15 min, and filtered through cloth. The extract was then concentrated in vacuo to the desired volume.

Different doses of extracts were injected intraperitoneally into groups of four mice. The number of deaths was counted at 48h after treatment. LD50 values and corresponding confidence limits were determined by the Litchfield and Wilcoxon method (PHARM/PCS Version 4).

The hot-plate test was assessed on groups of eight male and female mice. The temperature of the metal surface was maintained at 55±0.2°C. The latency to a discomfort reaction (licking paws or jumping) was determined before and after drug administration. The cut-off time was 25 sec.[23]

Thirty minutes after the administration of the extracts to groups of eight male and female mice, they were injected intraperitoneally with 0.7% v/v acetic acid solution (volume of injection 0.1 ml/10 g body wt.). The number of writhings produced in these animals was counted 5 min after acid injection for 30 minutes.[24]

The anti-inflammatory activity against acute inflammation was tested using by xylene-induced ear edema method in mice. Mice were divided into groups of eight. Thirty minutes after i.p. injection of the different doses of extract, diclofenac and 0.03 ml of xylene were applied to the anterior and posterior surfaces of the right ear. The left ear was considered as control. Two hours after xylene application, mice were sacrificed and both ears were removed. Circular sections were excised, using a cork borer with a diameter of 9 mm, and weighed. The increase in weight caused by the irritant was measured by subtracting the weight of the untreated left ear section from that of the treated right ear section.[25]

The anti-inflammatory activity against chronic inflammation was tested using cotton pellet granuloma method in mice. The pellets of dentistry cotton weighing 30 mg each were sterilized in an air oven at 121°C for 20 min and impregnated with 0.4 ml of an aqueous solution of ampicillin. Under ketamine (65 mg/kg body wt.) and xylazine (6.5 mg/kg body wt.) anesthesia, two cotton pellets were implanted subcutaneously in the shoulder region of mice, one on each side. The extract and diclofenac were given once daily for 7 days. On Day 8, the rats were killed and the pellets and surrounding granulation tissue were dried at 60°C for 24 h. The weight of granuloma was determined.[24]

The data were expressed as mean values ±SEM using SPSS software (version 15, Chicago, IL, USA) and tested with analysis of variance followed by the multiple comparison test of Tukey–Kramer. Discrepancies with P< 0.05 were considered significant.

## Results

The intraperitoneal LD50 values of J. regia aqueous and ethanolic leaves extract in mice were 5.5 g/kg (4.1- 6.5) and 3.3 g/kg (3.1- 3.5), and the maximum non-fatal doses were 4.1 g/kg and 2.93 g/kg, respectively.

**Fig. 1:**
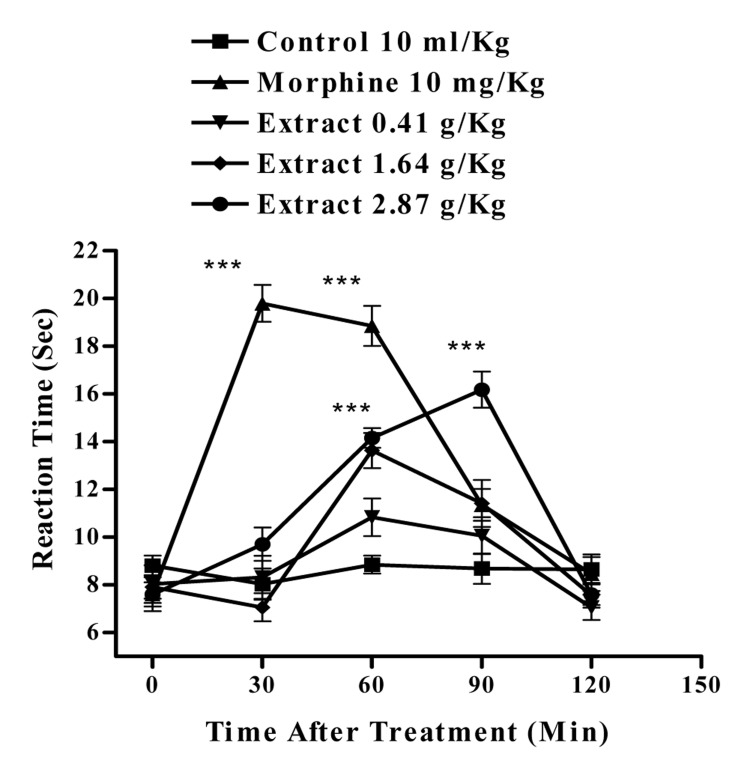
Effect of the aqueous extract of Juglans regia leaves and morphine on the pain threshold of mice in the hot-plate test. Each point represents the mean±SEM of reaction time for n=6 experiments on mice. ***P< 0.001, Tukey–Kramer test, Compared to control (saline)

**Fig. 2:**
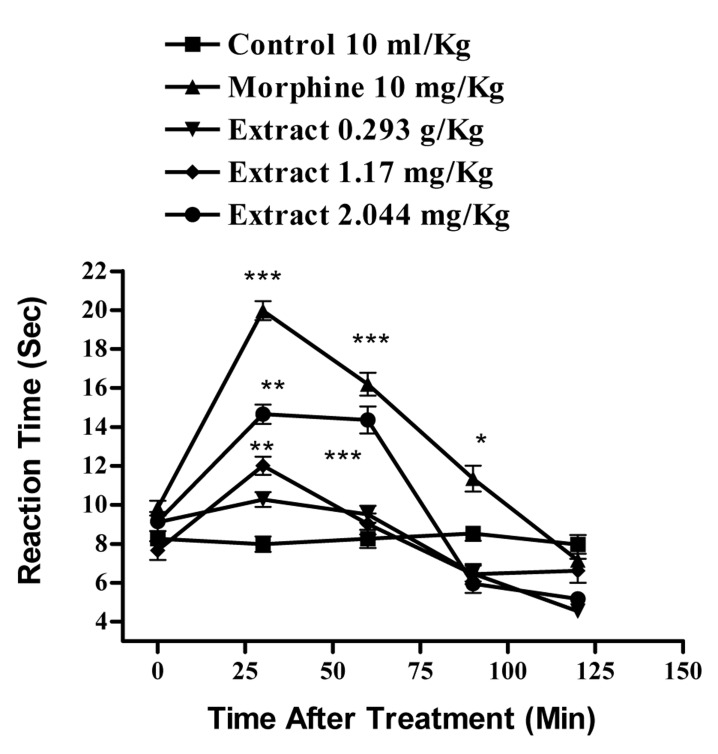
Effect of the ethanolic extract of Juglans regia leaves and morphine on pain threshold of mice in the hot-plate test. Each point represents the mean±SEM of the reaction time for n=6 experiments on mice. ***P< 0.001, **P< 0.01, *P< 0.05, Tukey–Kramer test, Compared to control (saline)

**Fig. 3:**
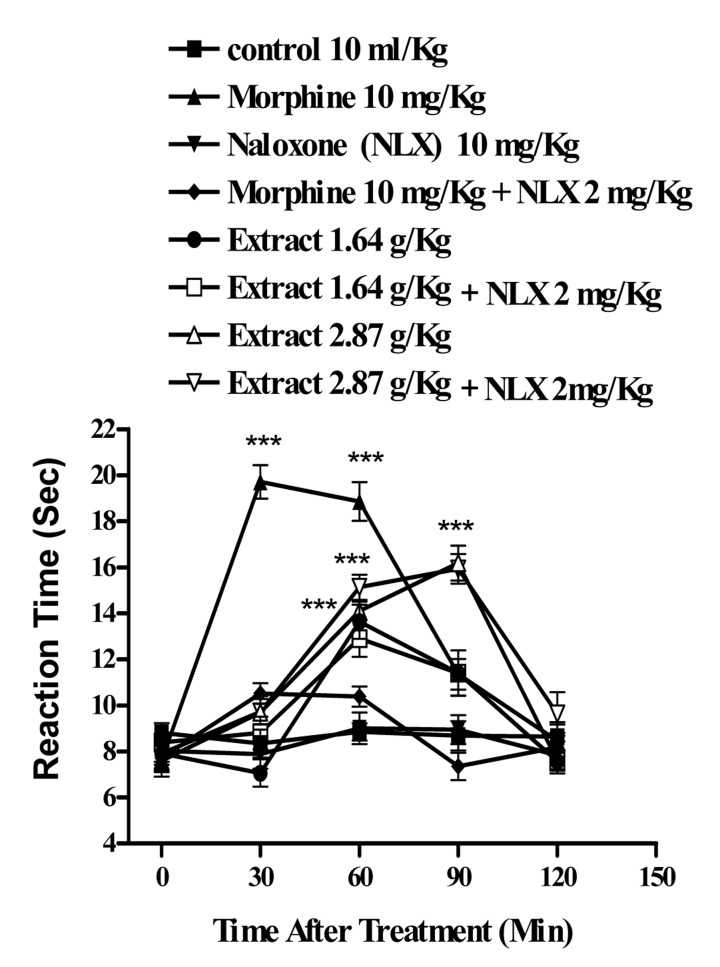
Effect of naloxone on the aqueous extract of Juglans regia leaves and morphine antinociceptive activity in mice using hot-plate test. Each point represents the mean±SEM of the reaction time for n=6 experiments on mice. Naloxone completely inhibited the effect of morphine and did not inhibit the effect of the extract. ***P< 0.001, Tukey–Kramer test, Compared to control (saline)

**Fig. 4:**
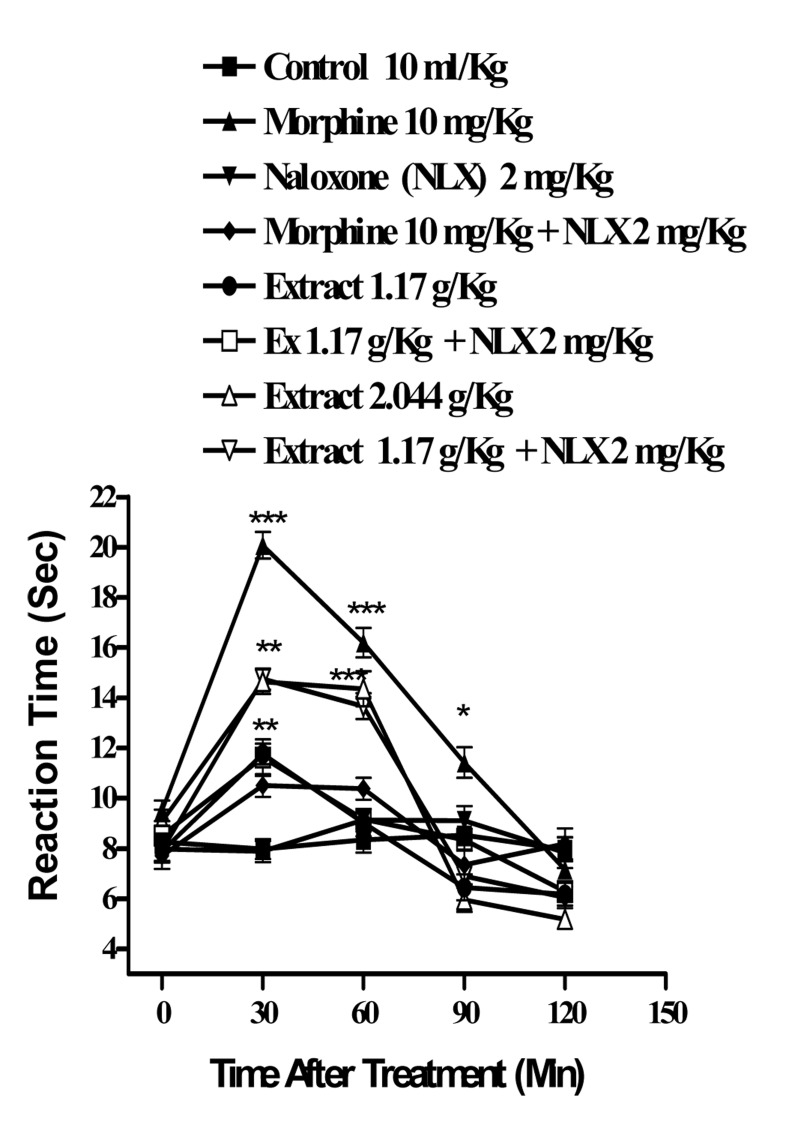
Effect of naloxone on the ethanolic extract of Juglans regia leaves and morphine antinociceptive activity in mice using hot-plate test. Each point represents the mean±SEM of the reaction time for n=6 experiments on mice. Naloxone completely inhibited the effect and morphine and did not inhibit the effect of the extracts. ***P< 0.001, **P< 0.01, *P< 0.05, Tukey–Kramer test, compared to control (saline)

In the hot plate test, the administration of the aqueous extract at doses of 1.64 and 2.87 g/Kg, (p<0.001) and ethanolic at doses of 2.44 g/Kg (p<0.001) and 1.17 g/Kg, (p<0.01) showed antinociceptive activity with duration of about 90-60 min, respectively. The time latency of the antinociceptive effect of high doses of both extracts was less than that of morphine (Figure 1 and Figure 2). Naloxone (2 mg/Kg., s.c.) pretreatment after i.p. injection of the extracts and morphine (10 mg/Kg), only inhibited the antinociceptive activity of morphine (p<0.001) (Figures 3 and 4).

**Fig. 5:**
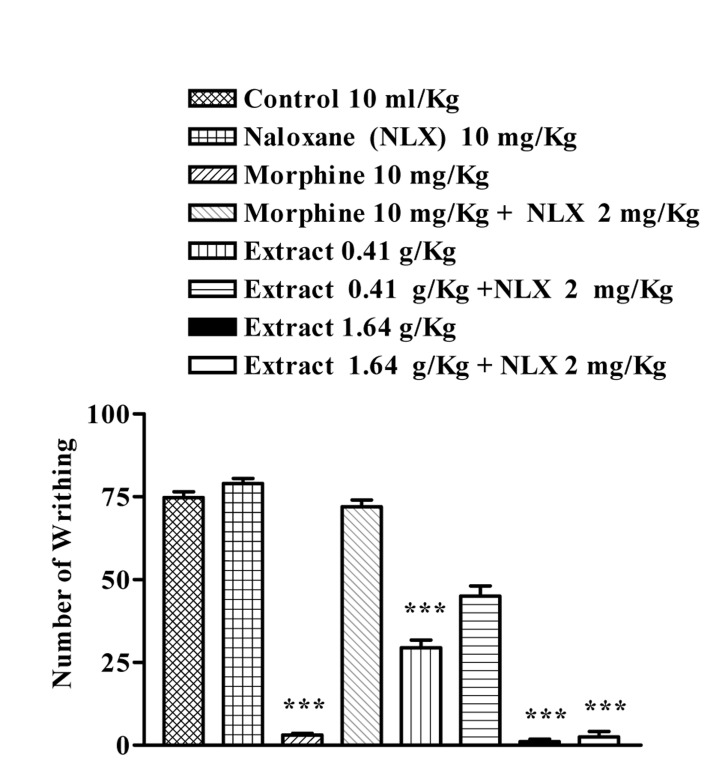
Effect of subcutaneously injection of naloxone on antinociceptive effect of the aqueous extract of Juglans regia leaves on acetic acid-induced writhing test in mice. The values are the mean±SEM for 7 mice. *** P< 0.001, Tukey–Kramer test, compared to control (saline)

**Fig. 6:**
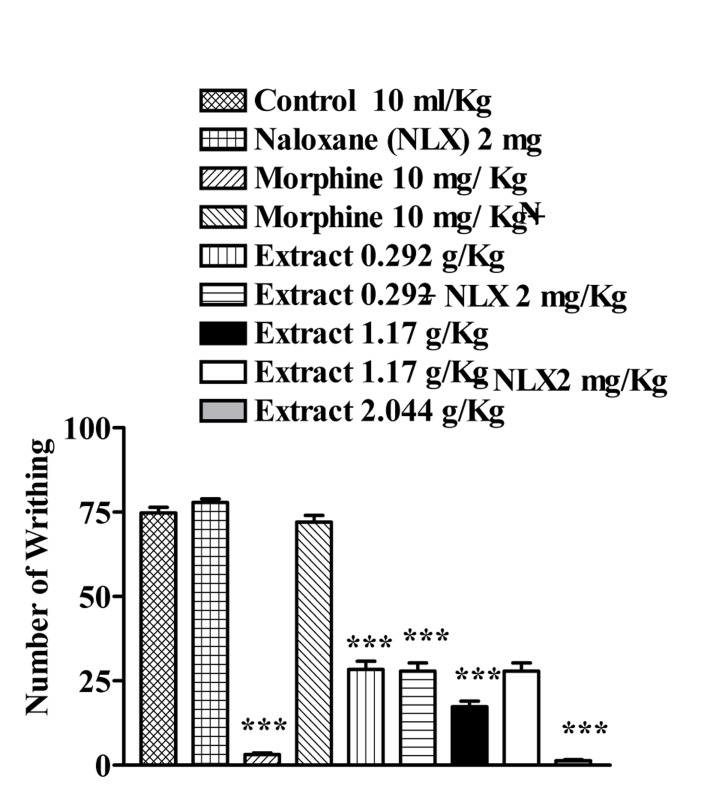
Effect of subcutaneously injection of naloxone on antinociceptive effect of the ethanolic extract of Juglans regia leaves on acetic acid-induced writhing test in mice. Values are the mean±SEM for 7 mice. *** P< 0.001, Tukey–Kramer, compared to control (saline)

The aqueous extract (0.41 and 1.64 g/Kg, P<0.001) and ethanolic extract (0.292, 1.17 and 2.44 g/Kg, p<0.001) of J. regia significantly reduced the number of mouse abdominal constrictions induced by a 0.7% acetic acid solution. Overall, naloxone (2 mg/kg, s.c.) pretreatment after i.p. injection of the extracts did not inhibit the antinociceptive activity of both extracts, p>0.05 (Figures 5 and 6).

In the xylene-induced ear edema study, the aqueous extract at a dose of 0.41 g/Kg, (p<0.01) and ethanolic extract at doses 0.292 g/Kg, p<0.01 and 1.17, 2.044 g/Kg, p<0.001) showed antiinflammatory activity that were not dose dependent (Table 1).

**Table 1:** Effect of the intraperitoneal injection of the aqueous and ethanolic extracts of Juglans regia leaves on xylene-induced ear swelling in mice^a^

**Treatment**	**Dose**	**Ear swelling (mg)**	**Inhibition (%)**
Control	10 ml/Kg	4.0±0.42	-
Diclofenac	15 mg/Kg	1.3±0.2^b^	67.5
Aqueous extract	0.41 g/Kg	1.76±0.6^b^	56
Aqueous extract	1.64 g/Kg	3.0±0.6	25
Aqueous extract	2.87 g/Kg	3.2±0.33	20
Ethanolic extra	0.292 g/K	2.0±0.27^c^	50
Ethanolic extra	1.17 g/Kg	1.1±0.31^c^	72.5
Ethanolic extra	2.044 g/Kg	2.5±0.4^b^	37.5

^a^ Values are the mean±SEM for 8 mice.

^b^ P< 0.01 and

^c^ P< 0.001 Tukey–Kramer, compared to control (saline).

In the chronic inflammation (cotton-plate) test, the aqueous and ethanolic extracts indicated anti-inflammatory effects and the aqueous extract showed maximum effects at a dose of 1.64 g/Kg, (p<0.001) and the maximum activity of ethanolic extract was observed at a dose of 1.17 g/Kg, (p<0.001) (Table 2).

**Table 2:** Effect of the intraperitoneal injection of the aqueous and ethanolic extracts of Juglans regia leaves (consecutive for 7 days) on the weight of granuloma in mice^a^

**Treatment**	**Dose**	**Cotton pellet (mg)**	**Inhibition (%)**
Control	10 ml/Kg	12.4±0.39	-
Diclofenac	15 mg/Kg	3.8±0.22^b^	69.35
Aqueous extract	0.41 g/Kg	10.3±0.41^b^	16.93
Aqueous extract	1.64 g/Kg	4.9±0.22^b^	60.48
Ethanolic xtract	0.292 g/Kg	11.6±0.66	6.45
Ethanolic xtract	1.17 g/Kg	8.9±0.33^b^	28.23

^a^ Values are the mean±SEM for seven mice.

^b^ P<0.001, Tukey–Kramer, compared to control (saline)

## Discussion

In the present study, the hot plate test and the acetic acid induced writhes in mice were selected to investigate the central and peripheral antinociceptive effects, respectively. The xylene-induced ear swelling in mice and the cotton pellet granuloma in rats were selected to present models of acute (exudative phase) and chronic (the poliferative phase) inflammation respectively.[25]

The present results indicate that aqueous and ethanolic extract of J. regia leaves have markedly central and peripheral antinociceptive activities. The extracts also showed activity against acute and chronic inflammation.

In respect to LD50 values, the ethanolic extract was more toxic than the aqueous extract. Compared with a toxicity classification,[26] these extracts are relatively toxic. The aqueous and ethanolic extracts showed antinociceptive activity in high doses in the hot plate test. The hot plate test is a specific central antinociceptive test.[27] The antinociceptive effect of the extracts was not inhibited by naloxone. Therefore, it is possible that the extracts exerted their effects through non-opioid receptors and the plant extract does not appear to be acting in the central nervous system through activation of opioid receptors. After injection of the extracts, sedative effects were observed in high doses which is possibility related to flavonol glycosides constituents like quercitrin and isoquercitrin.[28][29] It is possible that the antinociceptive effect was shown in high doses in the hot plate test that might be due to its sedative effects.

The antinociceptive activity of opioid agonists, opioid partial agonist, on non-steroidal anti-inflammatory agents can be determined using the writhing test.[24] The results obtained in this test and efficacy of both extracts suggest that these extracts possess peripheral analgesic properties. The antinociceptive activity of the extracts was not inhibited by naloxone, therefore the mechanism of action such as inhibition of cyclo-oxygenase probably was considered.

In xylene induced ear edema test, mediators of inflammation are released following stimulation. This leads to the dilation of arterioles and venules and may increase vascular permeability.[24]

The aqueous and ethanolic extracts showed anti-inflammatory effects in acute inflammatory tests with different efficacy in these tests. This plant may have a membrane-stabilizing effect that reduces capillary permeability and/or has inhibitory effects on the release of mediators. In higher doses, the anti-inflammatory efficacy, especially for the aqueous extract, was decreased. This might be related to some constituents in the extracts that oppose against anti-inflammatory activity.

The extracts reduced cotton pellet-induced granuloma, thereby suggesting its activity in the proliferative phase of the inflammation. Other studies have demonstrated that various flavonoids such as quercetin, luteolin, hesperidin produce significant antinociceptive and/or anti-inflammatory activities.[15][30][31][32][33][34][35] Therefore, it could be suggested that the antinociceptive and anti-inflammatory effects of the the aqueous and ethanolic extract of J. regia leaves may be due to their contents of flavenoids.

It is concluded that the aqueous and ethanolic extracts have central and peripheral antinociceptive effects. The non-opioid receptors or inhibition of cyclo-oxygenase enzyme may mediate these effects. The extracts showed also activity against acute and especially chronic inflammation. The extracts of J. regia could be considered as a promising analgesic and anti-inflammatory agents against diseases such as rheumatoid arthritis.
